# Enhancing Thoracic Surgery with AI: A Review of Current Practices and Emerging Trends

**DOI:** 10.3390/curroncol31100464

**Published:** 2024-10-17

**Authors:** Mohamed Umair Aleem, Jibran Ahmad Khan, Asser Younes, Belal Nedal Sabbah, Waleed Saleh, Marcello Migliore

**Affiliations:** 1 College of Medicine, Alfaisal University, Riyadh 11533, Saudi Arabia; 2 Thoracic Surgery & Lung Transplant, Lung Health Centre, Organ Transplant Center of Excellence (OTCoE), King Faisal Specialist Hospital & Research Center, Riyadh 11211, Saudi Arabia; 3 Minimally Invasive Thoracic Surgery and New Technologies, Department of General Surgery & Medical Specialties, University Polyclinic Hospital, University of Catania, 95131 Catania, Italy

**Keywords:** thoracic surgery, AI, artificial intelligence, VATS, RATS, precision medicine, precision surgery

## Abstract

Artificial intelligence (AI) is increasingly becoming integral to medical practice, potentially enhancing outcomes in thoracic surgery. AI-driven models have shown significant accuracy in diagnosing non-small-cell lung cancer (NSCLC), predicting lymph node metastasis, and aiding in the efficient extraction of electronic medical record (EMR) data. Moreover, AI applications in robotic-assisted thoracic surgery (RATS) and perioperative management reveal the potential to improve surgical precision, patient safety, and overall care efficiency. Despite these advancements, challenges such as data privacy, biases, and ethical concerns remain. This manuscript explores AI applications, particularly machine learning (ML) and natural language processing (NLP), in thoracic surgery, emphasizing their role in diagnosis and perioperative management. It also provides a comprehensive overview of the current state, benefits, and limitations of AI in thoracic surgery, highlighting future directions in the field.

## 1. Introduction

**Figure 1:**
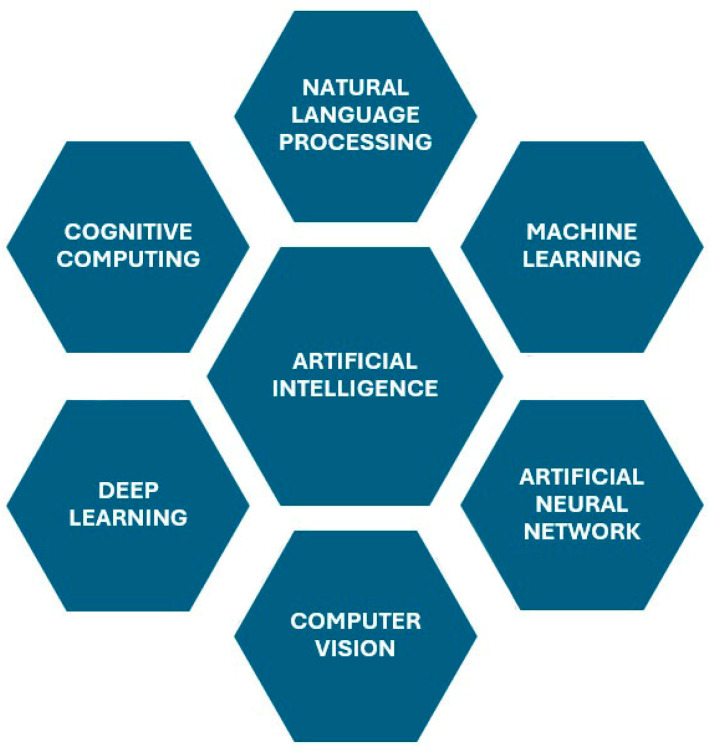
Subcategories of artificial intelligence [5].

**Figure 2:**
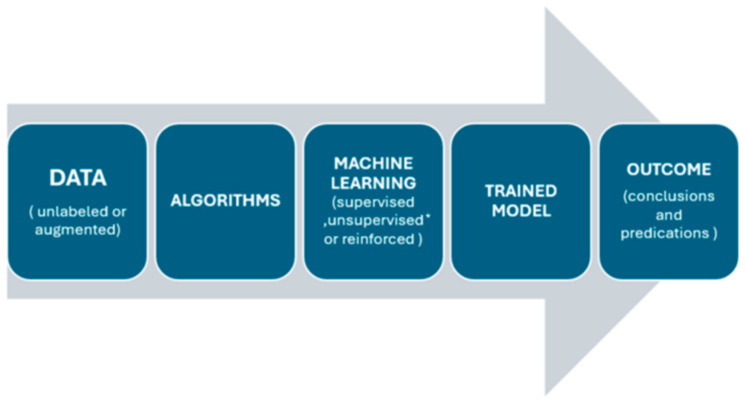
Process and steps in artificial intelligence [5]. * *Unsupervised methods are used for pattern recognition and clustering rather than generating predictive models*.

Artificial intelligence is increasingly becoming an integral part of medical practice, improving patient and healthcare team outcomes, lowering costs, and influencing public health [1]. Surgery is predicted to be impacted by current advancements in AI; however, due to several drawbacks, it has not yet reached its full potential in the discipline. The amount of data generated today outpaces the human ability to handle it cognitively, and AI is poised to play a significant role in supporting the provision of tailored healthcare (Figure 1 and Figure 2). For instance, current advances in AI have demonstrated great levels of accuracy in imaging and signal detection tasks. AI technologies in thoracic surgery are proving to be valuable in precise diagnoses, improving treatment planning, and optimizing postoperative care mainly using machine learning (ML) and natural language processing (NLP) techniques [2]. NLP is revolutionizing how clinical information is processed and analyzed. Additionally, ML algorithms are increasingly being used for in-depth analysis of imaging data, aiding in the lung cancer diagnosis and classification [3]. These result in numerous advantages, including reduced medical errors and quicker, more efficient patient care [3,4]. Certain challenges, however, have been noted, some of which include limited generalizability, data privacy concerns, the need for well-annotated and homogenous datasets for training, potential biases, and ethical considerations [2]. This article explores the current state of AI in thoracic surgery, covering diagnosis, operative planning, and postoperative management, while highlighting AI’s potential advantages and limitations.

## 2. Methodology

A comprehensive search was performed using the PubMed/MEDLINE, Cochrane, and Google Scholar databases. The articles were then screened for relevant studies by reviewing their abstracts with the following criteria: (1) topics: AI in healthcare and surgery, AI and respiratory medicine, AI and pathology, and AI and thoracic surgery; (2) published in English and in a peer-reviewed journal. Next, two authors (M.U.A. and J.A.K.) comprehensively reviewed the full manuscripts for inclusion. There was no year limit. The inclusion criteria prioritized but were not limited to clinical trials and meta-analyses on the role of artificial intelligence in thoracic surgery published from 2021 onwards. Articles of interest that had been cited by the articles identified in the initial search were also reviewed.

## 3. Discussion

### 3.1. Machine Learning and Natural Language Processing Applications in Thoracic Surgery

Thoracic surgery practice can be greatly aided by ML and NLP models, which aid in the diagnostic analysis of common and widespread diseases like non-small-cell lung cancer (NSCLC). About 84% of lung cancer cases are NSCLC, posing a great burden on healthcare, and is one of the greatest risks to human health due to its low 5-year relative survival rate of 25.0% [6]. The staging of NSCLC poses a great challenge to pulmonologists and thoracic surgeons because of the limitations of invasive modalities such as mediastinoscopy and ultrasound-guided transbronchial needle aspiration [7]. While such modalities have better diagnostic capabilities over non-invasive modalities such as CT and PET scans, they are not routinely used in screening or in clinical practice on patients with severe comorbidities [8]. Researchers have investigated employing statistical analyses or machine learning techniques to acquire nontrivial knowledge on full patient attributes and lymph node metastasis status in order to achieve exact staging [8,9,10,11,12,13,14,15]. The majority of clinical data, including tumor size, lymph node, tumor density, pleural indentation, and other information, are recorded in free-text format in electrical medical records (EMRs), which makes it difficult to manually extract data. Manual extraction takes a lot of time and is prone to human error. Thus, one major issue is how to efficiently extract these data to aid in later tasks like LNM prediction [16]. There has been an upsurge in the use of NLP models to extract this information automatically. Unfortunately, to date, this has not been widely adopted in thoracic surgery. In contrast, some medical fields have successfully implemented this technology. For instance, Chen et al. [17] computed the Cancer of the Liver Italian Program score by extracting data from various clinical notes, such as CT reports and operation notes. To determine the TNM and clinicopathological stage of colorectal cancer in Australian patients, Martinez et al. [18] took data from pathology records. To evaluate patients’ chances of survival, Yuan et al. [19] extracted several features from EMRs using NLP methods. Another recent study by Hu et al. [7] developed a lymph node metastasis prediction model for patients with NSCLC by integrating NLP and ML with EMR systems. They concluded that every machine learning model in their study outperformed the clinician’s assessment and the size requirements. Their experimental results also demonstrated that the NLP model can effectively extract information from CT reports, aiding in the lymph node metastasis prediction model’s development and updates, thereby facilitating its use in clinical settings.

Other areas where ML models have rapidly been adopted and show potential in thoracic surgery are diagnostic imaging and predictive analysis of surgical outcomes. These models will serve as a great addition for surgeons to foresee the surgical outcomes in patients undergoing high-risk surgeries or in patients with severe comorbidities. Kunze et al. [20] evaluated ML algorithms’ ability to predict clinically significant outcomes after orthopedic surgery and found that the currently available algorithms can easily discriminate the propensity to achieve clinically significant outcomes using the minimal clinically important difference with fair-to-good performance as evidenced by C-statistics ranging from 0.6 to 0.95 in most of the analyses. Another study by Stam et al. [21] found that AI algorithms can precisely predict surgical complications in major abdominal surgeries, provided they are thoroughly tested and validated and provided they are relying on a complete, balanced database. A 2023 study by Rana et al. [22] highlighted MRI and X-ray imaging as key imaging modalities for surgical disease detection using ML and DL techniques, with MATLAB and SVM as commonly used tools. Convolutional neural networks and random forest were found to outperform other algorithms, suggesting that the use of DL models with denoising approaches can improve accuracy. A common glossary of artificial intelligence is reported in Table 1.

### 3.2. Advanced AI Applications in Thoracic Surgery

#### 3.2.1. Identification of High-Risk Patients

Predictive analyses for the identification of high-risk patients are one of the most common applications of artificial intelligence. Several supervised and unsupervised ML models are utilized for the purpose of predicting binary events like readmission and mortality. A few examples of such incentives are the risk stratification index (RSI) [25], the American College of Surgeons National Surgical Quality Program (ACS NSQIP) [26], the Revised Cardiac Risk Index [27,28], and the Preoperative Score to Predict Postoperative Mortality [29]. An artificial neural network (ANN) method was created using genome sequencing data to guide safer and more successful warfarin dosing; in patients with international normalized ratios (INRs) > 3.5, the algorithm predicted the therapeutic dose with an accuracy of 83% [30]. For instance, an ANN-based model used to stratify postoperative bleeding risk in patients undergoing cardiac pulmonary bypass had a 92% accuracy rate [31]. To forecast the requirement for prolonged ventilation during coronary bypass grafting (AUC = 0.71–0.73), another ANN algorithm was created [32]. Patient safety could be significantly improved in these circumstances with early intervention. Other predictive models have deployed ML techniques for the purpose of estimating the postoperative discharge destination, down to the floor, within 24 h of surgery [33]. To increase these models’ predicted accuracy, full and real-time patient data are an absolute necessity.

#### 3.2.2. Predicting and Detecting Complications

AI-powered sensors and continuous monitoring would play a massive role in aiding a thoracic surgeon in the early detection of complications. Thoracic surgery involves several postoperative pulmonary complications, such as respiratory function and infections that frequently develop after major surgery, adding to the mortality rates, prolonged hospital stays, and increased costs [34,35,36]. Moreover, early-stage treatment of lung disorders may decrease the probability of bad outcomes, further emphasizing the need for early detection. To minimize such complications and aid in early detection, AI can assist in the development of prediction algorithms and decision support systems, while novel sensors and ongoing monitoring can aid in the collection of substantial volumes of physiological or electronic health record data [37]. The most frequently utilized classical model in thoracic surgery is the Assess Respiratory Risk in Surgical Patients in Catalonia (ARISCAT) score. ARISCAT was developed by Canet et al. [38] to foresee PPC in surgical patients using logistic regression (LR), which is the only score scale that maintains discriminator power for external acknowledgment [39]. The ARISCAT score attained an AUROC of 0.80 in a prospective validation study with 5859 patients [39]. Furthermore, Bolourani et al. [40] created an ML model to predict respiratory failure following pulmonary lobectomy in 4062 patients. The AUROC was not given, even though the sensitivity and specificity were 83.3% and 94.5%, respectively. Chen et al.’s study [41] examined several machine learning methods, such as LR, SVM, RF, adaptive boosting, and GBM, to predict the risk of pneumonia in 786 patients following orthotopic liver transplantation. Their study identified 14 factors, including laboratory and clinical variables, that were linked to postoperative pneumonia. Furthermore, opioid-induced respiratory depression frequently occurs in the postoperative general care unit. Early diagnoses of such respiratory episodes are now possible due to new portable and wearable monitoring technologies [42,43,44]. Since these events occur way before true code-blue events, early identification and intervention may provide an opportunity to avert disastrous consequences [45]. Score scales such as Prediction of Opioid-Induced Respiratory Depression in Patients Monitored by Capnography are the first step in the prediction of the risk of respiratory depression, using multivariable regression modeling on continuous oximetry and capnography data, followed by the next step, pattern detection with DL techniques [46].

Timely and effective treatment can make the crucial difference between saving a life and losing it. Such crucial calls can sometimes be mishandled by the surgeon in charge, but AI-driven decision support systems may help prevent these errors. Even during routine clinical care, interventions based on high-quality evidence are routinely miscalculated and not provided. Such high-quality evidence and patient data can be combined by a decision support system to provide point-of-care recommendations. For instance, Joosten et al. [47] demonstrated a positive effect on neurocognitive recovery by precisely titrating liquids, analgesics, and anesthesia using three closed-loop devices. AI can assist in the development of professional societies’ guidelines and recommendations under a lack of high-quality evidence. It can also assist in the development of guideline-based decision support systems. Using decision assistance and image-based navigational tools, hospitals can address inefficiencies and clinical issues faced by physicians during surgery by implementing AI and predictive analytics in conventional ORs [37]. Certain AI systems can assist in anticipating complications, ensuring smoother surgeries and quicker recovery by assessing the probability of problems even before a patient is transferred into the operating room. AI is already proving to be an extremely important aid by improving the identification of target areas, such as through the OR "black box" platform, which records and analyzes surgical procedures to identify potential issues. For instance, one hospital discovered frequent OR door openings during surgery due to the suture cart being outside the room, leading to its relocation back inside [37]. Complex OR environments can benefit from digitalization and AI integration, as seen with the Triton system, which uses AI and infrared technology to analyze sponge photos and quantify blood loss [48].

#### 3.2.3. Perioperative Management

Artificial intelligence can serve as a potent tool in perioperative thoracic surgery management by enhancing diagnostic processes, performing tumor staging, facilitating predictive analysis, enabling robotic-assisted surgeries, and reducing postoperative complications and mortality rates. Rapid Diagnostic Assessment and Tumor Staging can make the difference between survival and death during preoperative management. A recent study conducted by Ye et al. experimented with a deep learning radiomics model to predict high-risk pathologic pulmonary nodules in patients through CT semantics [49]. Their proposed combined model yielded area-under-the-receiver-operating-characteristic-curve (AUC) values of 0.872 and 0.814 in the training and external validation cohorts, respectively. Decision curve analysis (DCA) assures that the proposed models provide a net benefit in predicting high-risk pathological pulmonary nodules early on, which helps in providing rapid aid [49]. Similarly, such radiomic and clinical features can be combined to form comprehensive models to predict recurrence risk in NSCLC patients. A study extracted a total of 1562 radiomic features and retained 29 features after feature selection [50]. A COX multivariate regression model determined the N stage as the independent risk factor for postoperative recurrence. The AUC values of the radiomic–clinical comprehensive model were 0.972 and 0.937 in the training and test cohorts, respectively; these values far surpass those of the standalone clinical and radiomic models [50]. Another study conducted by Blüthgen et al. evaluated CT-derived radiomics for the thymic-epithelial-tumor stage and the presence of myasthenia gravis (MG) and concluded its usefulness as an imaging biomarker for thymic-epithelial-tumor histology and TNM staging [51]. Deep learning models’ efficiency in determining tumor stages was further cemented by two other studies conducted by Chen et al. [52,53], where both studies, respectively, dwelled on the use of CT image analytics through machine learning models for the identification of lung adenocarcinoma and high-risk tumor areas. The promising results from their computational study confirm that such a method provides a reliable basis for adenocarcinoma diagnoses supplementary to pathological examinations.

Intraoperative surgical management has also been flooded with artificial intelligence and deep learning model uses. Predictive analyses and robotic assistance throughout treatment have provided a massive upper hand to surgeons, allowing them to perform procedures with ease and smoothness. A deep learning prognostic model could assist in predicting the need for additional intraoperative placement of chest drainage after thoracoscopic lobectomy to prevent the risk of tension pneumothorax [54]. It was found that the incidence risk of tension pneumothorax was 4.53%, and the nomogram makes it possible to decide on the intraoperative installation of an additional pleural drainage tube and prevent complications associated with postoperative lung collapse. Prolonged air leakage is a serious and frequent complication post lung resection surgery. AI was used to develop a useful risk predictor model to help recognize patients who might benefit from additional preventive procedures, and the model was successfully able to confirm significant prognostic risk factors and protective factors for prolonged air leakage [55]. However, after internal validation, the C-statistic value was found to be 0.63, which is too low to generate a reliable score in clinical practice [55]. The use of AI in intraoperative planning and surgical management remains a field for further study and development.

Artificial intelligence (AI), machine learning (ML), and deep learning models are all equally transformative in postoperative surgical management in thoracic patients. Continuous monitoring of patients for predicting future potential complications is a major leverage point of AI for a thoracic surgeon. In a recent study conducted by Kadomatsu et al. [56], an elastic-net regularized generalized linear model that calculates a novel comorbidity risk score built especially for lung resection procedures was created. Data from 2018 and 2019 were used to validate this model, which examined 40 variables such as surgical methods, tumor-related characteristics, and patient status. The final 20-variable model performed noticeably better in predicting postoperative complications, with an AUC of 0.734, as opposed to 0.521 for the Charlson Comorbidity Index (CCI). Furthermore, AI models have been significantly developed for other critical care settings. For instance, a study by Frades et al. [57] aimed to create a mortality predictor for Intermediate Respiratory Care Unit (IRCU) patients using machine learning models. In the first year of implementation, the resulting model’s combination with multivariate logistic regression analysis considerably lowered failure rates by 50%, indicating its efficacy in mortality risk detection and real-time monitoring. As AI technologies continue to evolve, their integration into postoperative care protocols will likely become increasingly sophisticated, further enhancing recovery and long-term health outcomes for thoracic surgery patients.

#### 3.2.4. Immunotherapy Guidance

AI has further emerged as a revolutionizing tool in immunotherapeutic guidance for thoracic surgery, particularly through model development for recognizing DNA methylation biomarkers, RNA sequencing, selecting effective drug targets, and gene classification. Deep learning AI models may be able to meet the unmet demand for non-invasive testing for the early diagnosis of lung malignancies by integrating imaging, clinical, and DNA methylation biomarkers to enhance the classification of pulmonary nodules [58]. In a prospective study conducted across 24 hospitals in China, He et al. [58] proposed a combined model, PulmoSeek Plus, which integrates clinical, imaging, and cell-free DNA methylation biomarkers to improve the classification of pulmonary nodules. The model achieved an AUC of 0.85 in the validation cohorts, outperforming the standalone CIBM and PulmoSeek models, and had a sensitivity of 0.98 for early-stage lung cancer and a DCA indicating a substantial reduction in unnecessary surgeries and delayed treatments. In addition, AI has highlighted the role of dendritic cells in lung adenocarcinoma. Zhang et al. [59] conducted a study on lung adenocarcinoma and numerous other cancer patients undergoing immunotherapy, identifying 83 DC marker genes; this led to the development of a seven-gene signature that predicts prognoses and treatment responses. It was found that patients with lower risk scores responded better to immunotherapy and targeted therapies, and Cathepsin H emerged as a new protective biomarker for lung adenocarcinoma [59]. Similarly, an eight-gene risk model was successfully developed to predict esophageal squamous cell carcinoma induced by the tricarboxylic acid (TCA) cycle [60]. The function assay suggested the CTTN gene’s role in cell proliferation and invasion through the EMT pathway and highlighted the importance of the TCA cycle’s importance in tumor immunity. The role of AI predictive and analytic models is further solidified by studies such as Li et al.’s [61], where cuproptosis was studied as a promising therapeutic modality for LUAD patients who had grown resistant to radiotherapy and chemotherapy. By identifying cuproptosis-related genes (CRGs) and constructing a three-layer ANN risk model, a robust prognostic value was demonstrated where low-risk group patients had immune "hot" tumors with anticancer activity and were more sensitive to immunotherapy, while high-risk group patients had immune "cold" tumors and were more sensitive to chemotherapy. Furthermore, another comprehensive study by Chen at al. [62] utilized machine learning techniques to develop an autophagy-related gene (ARG) classifier based on eight key ARGs. This classifier showed excellent diagnostic and prognostic performance (AUC > 0.85) across multiple datasets, outperforming traditional markers like procalcitonin and C-reactive protein. The model also correlated significantly with immune cell infiltration, immune pathways, and cytokine levels, effectively reflecting the immune microenvironment during sepsis [62]. Areas of AI applications in thoracic surgery are demonstrated in Table 2.

### 3.3. Limitations and Ethical Implications of Utilizing AI

While AI can have numerous advantages, as we highlighted, it also comes with its risks [64].

Regardless of the extent to which ML is employed, a robot is believed to be unable to achieve fully autonomous thoughts. It is assumed that robots consistently replicate human cognitive processes, albeit with greater speed and logical consistency. As a result, human intuition and experience remain crucial factors. Surgeons frequently rely on instinct, and AI will most likely not be able to replace a human approach, at least not fully yet [64]. Another major limiting factor for using AI in surgery is the financial burden of operating such advanced technology. Generalization and uniform standards of treatment will be impaired because not all regions can afford the technology. It is possible that an algorithm created in one institution will not apply to other universities directly. The model will be effectively tailored to reflect the clinical experience of that institution through the development of the algorithm. This presents advantages as well as disadvantages for prospective consumers [65]. The quantity and quality of the data that are provided determine how well AI responds. The degree to which AI algorithms may be applied to different subgroups depends heavily on a number of criteria, including outliers, missing data, and how representative the included populations are. In fact, it is critical to regularly update algorithms with fresh patient data so that they can adjust how decisions are made [66]. The types and quality of the data that are accessible to the algorithm restrict its outputs; for example, lung cancers that affect Caucasians in the EU do not share the same epidemiologic features as lung cancers that affect persons in Asia. Nonsmoking reasons account for a larger percentage of cancer cases in Asia, especially in women [67]. Therefore, there is a chance that selection bias will affect projections if specific populations or sexes are underrepresented.

Moreover, most of the uses of AI-driven technology in surgery are yet in their infancy stage. The autonomous execution of complicated surgical procedures is not the same as that of simple independent tasks. ML is not always accurate and may produce inaccurate results, and hence, it is a must to overview whatever decisions the AI software may suggest. IBM’s Watson for Oncology Cognitive Computing system, created in 2012, employs AI algorithms to provide therapy suggestions for a range of illnesses, including lung tumors. Oncologists at the Memorial Sloan Kettering Cancer Center (New York, NY, USA) trained the program to recognize important information related to a patient’s cancer, such as blood test results, pathology and imaging reports, and the existence of genetic mutations. The available treatment options broadly align with the standards of the National Comprehensive Cancer Network [66]. However, in 2018, IBM’s Watson faced criticism for making incorrect treatment recommendations in certain situations, potentially endangering the lives of patients [68]. It is strictly contraindicated for a patient with lung squamous cell carcinoma to use bevacizumab, as recommended by the system. In June 2019, the American Society for Clinical Oncology received an abstract about IBM’s Watson for Oncology Cognitive Computing system, which hinted at its potential to support multidisciplinary tumor boards’ decision-making [69]. Another example backing this argument is a study that attempted to use ML for early pneumonia diagnoses: two sets of chest radiographs—one with pneumonia and the other without—were given to the algorithms so they could learn to distinguish between the two. The mark on the radiograph used to designate the right and left sides, which turned out to differ across the two hospitals, was the greatest predictive feature of pneumonia, according to the algorithm, which immediately determined this [70]. Because of this, ML still requires supervision at this point in its development.

To fully achieve the potential of AI in thoracic surgery, some key ethical concerns must be tackled, including obtaining informed consent for data use, ensuring safety and transparency, addressing fairness and biases in algorithms, and safeguarding data privacy [71]. Overall, the legality of ML models also remains debated (Resolution of the European Parliament, 16 February 2017) [72]. The primary motive is to counteract the ethically challenging circumstances brought about by implementing AI in thoracic surgery [73]. Most legal discussions around artificial intelligence have focused on the issue of algorithmic transparency. The use of AI in high-risk scenarios emphasizes the need for transparent, fair, and responsible design, with essential components being clarity and accessibility [74]. It is common for information regarding the operation of algorithms to be purposefully difficult to access [75]. Machines that can learn new behavioral patterns and function according to lose norms are said to pose a danger to our ability to assign blame to their creators or operators. We might not have anyone to hold responsible for any harm caused if AI is used [74,75]. The scale of the threat is uncertain, and using machines will drastically restrict our capacity to assign responsibility and take charge of the decision-making process [76]. Modern computing approaches can obscure the reasoning behind the outputs of an AI system, making it difficult for non-technical clinical users to understand [77]. While AI systems like IBM’s Watson for Oncology are designed to support clinical decision-making by evaluating information and recommending patient care, the complexity of these systems can create challenges [74,75]. If widely adopted, AI systems could revolutionize clinical decision-making and reshape healthcare dynamics, making it crucial for clinicians to ensure the safe implementation of these technologies [78,79,80]. However, as this study tries to clarify, it is possible that AI will be able to get beyond these restrictions; at the moment, one can only hypothesize as to how AI will be able to get around some ethical and moral conundrums. A summary of the key topics discussed in the above paragraphs regarding the identification of high-risk patients, predicting and detecting complications, perioperative management, and immunotherapy guidance is highlighted in Table 3.

## 4. Conclusion

Artificial intelligence (AI) has shown the potential to revolutionize thoracic surgery by enhancing diagnostic accuracy, optimizing treatment plans, and improving postoperative care. AI models, such as those predicting lymph node metastasis in non-small-cell lung cancer, have demonstrated comparable performance over traditional methods, aiding in precise staging and better clinical decision-making. Moreover, AI-driven robotic surgeries, perioperative intelligence, and predictive models for identifying high-risk patients and complications have paved the way for safer and more efficient surgical procedures.

Despite these advancements, however, the integration of AI in thoracic surgery faces some challenges, including the need for extensive datasets, problems of data privacy, potential biases in AI models, and ethical concerns. Addressing these limitations requires continuous refinement of AI models, adherence to ethical guidelines, and the establishment of transparent and accountable AI systems. Looking ahead, the future of AI in thoracic surgery holds promise; there is the potential for more sophisticated AI-driven systems, advanced perioperative management models, and decision support systems that can integrate seamlessly into the clinical environment. More research and external validation will be essential to harness AI’s full potential and ensure its effective application in thoracic surgery.

## Figures and Tables

**Table 1:** Common glossary of artificial intelligence [23,24].

Algorithm	Models’ Creation from Mathematical Approaches to Data
Model	A mathematical function produced by an algorithm using a training set of data.
Artificial intelligence	A branch of computer science that focuses on teaching machines to perform tasks that traditionally require human intellect.
Machine Learning	A subfield of artificial intelligence where computers can learn from experience and recognize patterns in data without the need for explicit programming.
Deep learning	An area of machine learning that uses multilayer neural networks to examine data and create representations that resemble human thought processes.
Supervised learning	A subdivision of ML where the algorithm learns from labeled training data for the purpose of the classification or prediction of new data.
Unsupervised learning	A subset of machine learning where computer algorithms are trained on unlabeled training data to find patterns and draw conclusions. *Unsupervised methods are used for pattern recognition and clustering rather than generating predictive models.
Computer vision	An artificial intelligence field where computers to identify, understand, and react to visual input data.
Natural language processing	An AI field that enables computers to understand, interpret, and react to voice or text input.

**Table 2:** Areas of AI applications in thoracic surgery [63].

Application Areas	Examples
Preoperative phase	Early detection of lung cancer; genetic review and decision-making in chemo- vs. immunotherapy
Diagnosis	Improving diagnostic accuracy and reducing false-positive rates in radiology, liquid biopsy, and histology
Operative phase	Enhancing surgical safety, accuracy, and decision-making in robotic-assisted surgery
	Surgical skill assessment
	Surgical planning optimization
Postoperative phase	Predicting complications and mortality risks post-surgery
	Enhancing risk stratification
	Supporting clinical decision-making
Education	Providing educational support and surgical training feedback
Management	Improving operating room scheduling and efficiency
	Optimizing overall resource utilization
	Enhancing cost-effectiveness

**Table 3:** Summary of key topics related to AI applications in thoracic surgery.

Section	No. of Studies	Main Outcomes	Advantages	Disadvantages
Identification of High-Risk Patients	7	Models accurately predict mortality, readmission, bleeding, and ventilation needs, e.g., 83% warfarin dose accuracy and 92% postoperative bleeding prediction.	Improved patient safety and outcomes through early intervention; high accuracy in stratifying risk.	Real-time data are required for higher prediction accuracy; generalization to all institutions is challenging due to the differences in the available data and resources.
Predicting and Detecting Complications	12	Successful use of ML for predicting respiratory failure, pneumonia, and opioid-induced depression; ARISCAT and OR "black box" systems showed utility.	Early identification of complications, real-time monitoring, and decision support; reduce surgical errors and improve patient outcomes.	Requires large datasets for validation; not always reliable when applied across diverse clinical settings. Computational costs may be prohibitive.
Perioperative Management	6	AI-assisted diagnosis (CT-based radiomics) improves tumor staging, and reduces complications (e.g., air leaks); DL models show AUC values of up to 0.97 for predicting recurrence and risk stratification.	Enhances diagnostic accuracy and intraoperative decisions; AI-assisted surgery improves precision and reduces complications.	Low external validation in some cases (AUC of 0.63 for some predictions); potential for low accuracy when generalizing models to different patient populations or regions.
Postoperative Management	4	Predictive models (e.g., elastic-net regularized models) improve the early detection of complications and reduce mortality; for example, 50% reduction in failure rates in IRCU patients.	Continuous monitoring and prediction of future complications; faster recovery and improved patient outcomes.	Data from select regions may not generalize well; updates to models are needed as well as new patient data to maintain accuracy.
Immunotherapy Guidance	6	Models predict biomarkers for immunotherapy (e.g., DC markers), classify pulmonary nodules, and enhance early cancer diagnosis, prognosis, and drug target identification.	Non-invasive testing; improved classification of cancer subtypes; personalized immunotherapy guidance with high predictive accuracy.	Limited validation across broader populations; potential issues with data privacy when using genetic markers and large-scale sequencing.
